# Micro-particle manipulation by single beam acoustic tweezers based on hydrothermal PZT thick film

**DOI:** 10.1063/1.4943492

**Published:** 2016-03-03

**Authors:** Benpeng Zhu, Jiong Xu, Ying Li, Tian Wang, Ke Xiong, Changyang Lee, Xiaofei Yang, Michihisa Shiiba, Shinichi Takeuchi, Qifa Zhou, K. Kirk Shung

**Affiliations:** 1 School of Optical and Electronic Information, Huazhong University of Science and Technology, Wuhan 430074, China; 2 State Key Laboratory of Transducer Technology, Chinese Academy of Sciences, Shanghai 200050, China; 3 Department of Biomedical Engineering and NIH Transducer Resource Center, University of Southern California, Los Angeles, California 90089-1111, USA; 4 Department of Physics and Key Laboratory of Acoustic and Photonic Materials and Devices of Ministry of Education, Wuhan University, Wuhan 430072, China; 5 Medical Engineering Course, Graduate School of Engineering, Toin University of Yokohama, Yokohama 225-8501, Japan

## Abstract

Single-beam acoustic tweezers (SBAT), used in laboratory-on-a-chip (LOC) device has promising implications for an individual micro-particle contactless manipulation. In this study, a freestanding hydrothermal PZT thick film with excellent piezoelectric property (d_33_ = 270pC/N and k_t_ = 0.51) was employed for SBAT applications and a press-focusing technology was introduced. The obtained SBAT, acting at an operational frequency of 50MHz, a low f-number (∼0.9), demonstrated the capability to trap and manipulate a micro-particle sized 10μm in the distilled water. These results suggest that such a device has great potential as a manipulator for a wide range of biomedical and chemical science applications.

The contact-free manipulation of micro-particle is of great use for the biological and medical fields. Hence, research on precise micro-particle positioning has attracted much attention recently. Dielectrophorestic^1^ and magnetic^2^ forces have been traditionally used to remotely manipulate micro-particles. Nevertheless, these two technologies are limited to polarizable particle and magnetic particle, respectively. To realize electrically neutral and nonmagnetic micro-particle contactless manipulation, optical tweezers has been developed.^3^ Unfortunately, the high energy of the focused laser often causes photo-damage in the targeted sample.^4^ In addition, the optical radiation force (at pico-newton level) is too small to manipulate larger particles. To address these issues, acoustic tweezers utilizing single beam,^5–8^ standing wave^9^ and surface acoustic wave^10,11^ have been proposed. As a promising single-particle manipulator, single beam acoustic tweezers (SBAT) is attractive. Its narrow acoustic beam, which has a steep intensity gradient that is produced using high-frequency ultrasound and a tight focus, can trap and manipulate micro-particle in a contact-free fashion, producing almost no damage.^7^ To form a highly focused acoustic beam, SBATs require a high-frequency ultrasound transducer with a low f-number (f^#^= focal length / aperture size);^6,12^ leading to that, the fabrication of SBAT remains challenging.

Piezoelectric thick films are regarded as a good candidate for high-frequency (≥50MHz) transducer applications;^13^ however, lapping down a piezoelectric single crystal or ceramic to a thickness of several tens of micrometers is both difficult and time-consuming. Almost all of the high frequency transducers based on piezoelectric thick films have been aimed at ultrasound imaging; in these cases, the transducer does not need to be tightly focused (f-number close to 1). To date, only ZnO films have been reported for SBAT applications.^14,15^ The main reason for this is that, when compared with other piezoelectric films, ZnO films are much easier to grow with a preferential orientation on different substrates; this property of ZnO films makes them suitable for the implementation of lens^14^ and self-focusing^15^ technologies to form steep acoustic beams. However, the weak piezoelectric response of ZnO (d_33_ ≈ 10–26 pm/V, k_t_ ≈ 0.28)^16^ presents a major obstacle, limiting the trapping force. Besides, this film sputtering technology is just right for ultra-high frequency (>100MHz) SBAT, and it doesn’t work for the SBAT with a relatively lower operational frequency, for example 50MHz∼100MHz. In our previous work,^17–19^ hydrothermal PZT thick films have been successfully used to fabricated 50MHz∼100MHz ultrasound transducer and array. It is worth mentioning that this kind of film exhibited a superior electromechanical coupling coefficient k_t_ = 0.52.^18^ The k_t_ value of a film represents its conversion efficiency between electrical and acoustic energy; generally, the higher the k_t_ value, the greater the trapping force will be. Hence, SBAT based on such hydrothermal PZT thick film is worthy to be expected.

This study focused on the feasibility of applying hydrothermal PZT thick films to produce SBAT. Because lens and self-focusing technologies are not suitable for hydrothermal PZT thick films, we investigated a press-focusing method. The manipulation of micro-particle was realized; the trapping force was discussed, and the trapping experiments were performed.

Using a novel rapid heating and cooling technology, a 35-μm hydrothermal PZT thick film was self-separated intactly from Ti substrate due to the different thermal expansion coefficients.^17^ As shown in Fig. 1(A), the obtained freestanding film was translucent, and had an area of 2 cm × 1.5 cm. After poling, the film’s d_33_ was measured to be 270pC/N, which is comparable to that of piezoelectric bulk material. The process of fabricating a tightly focused, high-frequency ultrasound transducer is illustrated schematically in Fig. 1(B)–1(E). First, E-Solder 3022 (Von Roll Isola Inc., New Haven, CT) was selected as the backing layer, and was then cast (using centrifugation) on an Au-coated hydrothermal PZT thick film. After the acoustic stack was diced into a 1.5 mm × 1.5 mm grid, it was tuned to a 1-mm diameter, and connected via a wire on the backing layer; it was then placed concentrically in a brass housing, using Epoxy 301 (Epoxy Technologies, Billerica, MA) as an insulator to fill the gap between the stack and the housing, as shown in Fig. 1(B). To obtain a highly focused acoustic beam, a special fixture, displayed in Fig. 1(C), was designed. Using this fixture, the transducer could be held with a stainless steel ball in its center during the press-focusing^20,21^ process; this process is depicted in Fig. 1(D) and 1(E). Afterward, an Au layer and a parylene layer were deposited on the front of the transducer (which had an f-number of 0.9) as the upper electrode and matching layer, respectively. Finally, the transducer was assembled with a SMA connector, which is shown in Fig. 1(F).

As illustrated in Fig. 2(A), it can be seen that resonant and anti-resonant peaks are located at 46.8MHz and 53.2 MHz, respectively. According to the IEEE standard,^22^ electromechanical coupling coefficient in thickness mode (k_t_) can be given by

$$k_{t}=\sqrt{\frac{\pi }{2}\frac{f_{r}}{f_{a}}\tan\left(\frac{\pi }{2}\frac{f_{a}-f_{r}}{f_{a}}\right)}$$ (1)

Substituting the appropriate values into Eq. (1), k_t_ is calculated to be 0.51, which is similar to our previous result.^18^ It is worthy to be noticed that no piezoelectric thick film has been reported to possess such high k_t_ value after being tightly press-focused. This may be attributed to the unique columnar microstructure of hydrothermal PZT thick film.^18^ Additionally, a peak in the phase curve is observed at 50.4MHz, indicating that the central frequency of the tightly focused thick film transducer is nearby. A pulse-echo test was conducted in a deionized water bath at room temperature, and an X-cut quartz plate was selected as the target. The measured pulse echo waveform and normalized frequency spectrum are shown in Fig. 2(B). The central frequency of the hydrothermal PZT thick film transducer is 50 MHz, in good agreement with the electrical impedance result. The bandwidth at −6 dB is tested to be 40%. Such narrow bandwidth is caused by no matching layer, but it has almost no impact for acoustic trapping.^6^

A commercial PVDF needle hydrophone (Precision Acoustics, UK) was used to measure the acoustic pressure of this highly focused transducer with a voltage of 30 Vpp.^6^ The experimental acoustic pressure distribution (black solid line) in the lateral direction is shown in Fig. 3(A). The acoustic pressure at a frequency of 50 MHz exhibited a Gaussian distribution, and had a maximum value of 0.8 MPa at the focus point. There is no experimental method available to evaluate the trapping force of SBATs at frequencies higher than 40 MHz; hence, simulations were used as an alternative. The finite-element analysis and solver software package Comsol Multiphysics was used. The results of the simulations (shown as the red solid line in Fig. 3(A)) agreed with the experimental results. The acoustic pressure field distributions at the focal plane are shown in Fig. 3(B) (x–y plane) and 3(C) (x–z plane).

The configuration used in the SBAT trapping experiment is described in Fig. 4(A). The highly focused transducer was set in a chamber filled with distilled water; the chamber had an acoustically transparent mylar film at the bottom of the opening. The transducer was mounted on a motorized three-axis positioner (SGSP 26-50, Sigma KOKI Co., Japan), and was controlled using customized LabVIEW programs. A CMOS camera (ORCA-Flash 2.8, Hamamatsu, Japan), in combination with an inverted microscope (IX-71, Olympus, Japan), was placed under the chamber, to record the motion of the trapped particles. For the experimental study, we selected 10-μm (diameter) polystyrene particles; the density, longitudinal wave velocity, and transverse wave velocity of these particles were 1050 kg/m,^3^ 2340 m/s, and 1100 m/s, respectively. In a single focused Gaussian beam, the acoustic radiation force can be evaluated by integrating the time-averaged radiation stress tensor $〈{\text{S}}_{T}〉$ over a surface enclosing the object:^23^

$$F=\int_{s}〈{\overset{↔}{S}}_{T}〉\cdot \text{d}A,$$ (2)

where the integral is carried over, and the whole surface of the particle and the differential area dA points to its outer normal. *S_T_* is the time-averaged Brillouin radiation stress tensor:

$$〈S_{T}〉=\left(\frac{\rho_{0}uu^{*}}{4}-\frac{\left|p^{2}\right|}{4\rho_{0}c_{0}^{2}}\right)I-\frac{\rho_{0}uu^{*}}{2},$$ (3)

with *ρ*_0_ (1000 kg/m^3^) and *c*_0_ (1540 m/s) being the static mass density and sound velocity of the surrounding fluid, respectively, and the asterisk denoting the usual complex conjugation. Here, *I* is a unit tensor, and *p* = − *iωρ*_0_*ψ* and *u* = − ∇*ψ* are, respectively, the first-order velocity and pressure fields, which are related via the velocity potential *ψ*, with angular frequency ω. Using the measured value of the incident sound pressure *p*, *ψ* and *u* could be derived; after substituting for p and u in the Brillouin stress tensor $〈{\text{S}}_{T}〉$, the acoustic radiation force on the PS sphere was obtained. Disregarding friction, there were four forces acting on the particle: gravity, buoyancy, the force resulting from the support provided by the mylar film, and the acoustic radiation force. The gravitational, buoyancy, and support forces were always in the axial direction. The acoustic radiation force, however, was related to the position of the particle in the single focused Gaussian beam, and could be decomposed into an axial radiation force and a transverse radiation force. In our experimental system, the total force on the polystyrene particle in the axial direction was always zero. A plot of the calculated transverse radiation force against the lateral distance away from focal beam center is shown in Fig. 4(B). It is worth noting that this force pointed to the focal beam center. At the focal beam center, no transverse radiation force existed, and the polystyrene particle was in a force equilibrium state. If a small lateral distance was produced between the particle center and the focal beam center by the movement of the transducer, a transverse radiation force was produced that pushed the particle back to the focal beam center. The transverse radiation force thus acted as the trapping force, the calculated value of which was on the order of nano-Newtons (nN).

Figure 5 clearly demonstrates the capability of the SBATs based on a hydrothermal PZT thick film to trap and manipulate a single polystyrene particle (Multimedia view). A red circle is given as a reference point to show the location change of the microparticle. The hydrothermal PZT thick film transducer was driven by a sinusoidal burst with parameters of an excitation frequency of 50 MHz, a driving voltage of 30 Vpp, a duty cycle of 0.2%, and a pulse repetition frequency of 1 kHz. A series of images were captured at a frame rate of 10 frames per second. It was observed that—with no contact between transducer and microsphere—a single 10-μm polystyrene particle (in the red circle) was manipulated according to the movement of the transducer up (B), right (C) and down (D). This result suggests that hydrothermal PZT thick films are a good candidate material for SBAT applications. Because the size of the trapped particle is at the cellular level, the SBATs based on a hydrothermal PZT thick film could act as a single-cell manipulator for a wide range of applications in the biomedical and chemical sciences.

## CONCLUSION

In summary, a 50-MHz, high-frequency ultrasound transducer with an f-number of 0.9 was obtained based on a hydrothermal PZT thick film with excellent piezoelectric property. The trapping force acting on a 10-μm microparticle in the acoustic microbeam produced by the obtained device was calculated to reach the nano-Newton level. This force led to the realization of contact-free microparticle trapping and manipulation in distilled water. Results from this study demonstrate that hydrothermal PZT thick films are a promising choice for the application of single-beam acoustic tweezers.

## Figures and Tables

**FIG. 1.**
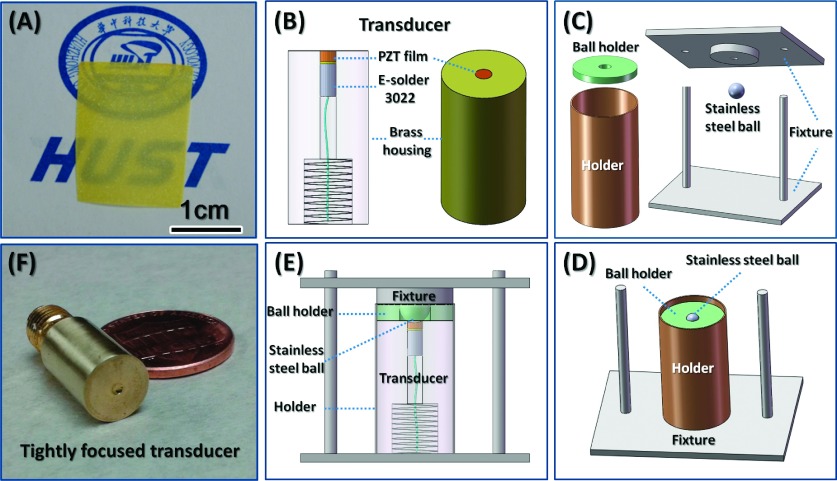
(A) Photograph of freestanding hydrothermal PZT thick film; (B)–(E) process used to fabricate a tightly focused high-frequency ultrasound transducer; (F) photograph of the transducer obtained for SBAT applications.

**FIG. 2.**
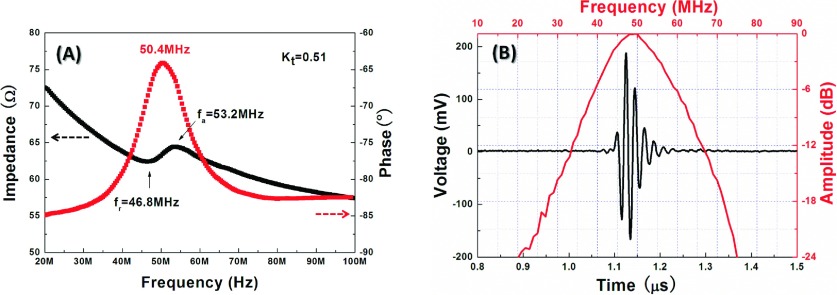
(A) Electrical impedance magnitude and phase of the transducer as a function of frequency; (B) The measured pulse echo waveform and normalized frequency spectrum.

**FIG. 3.**
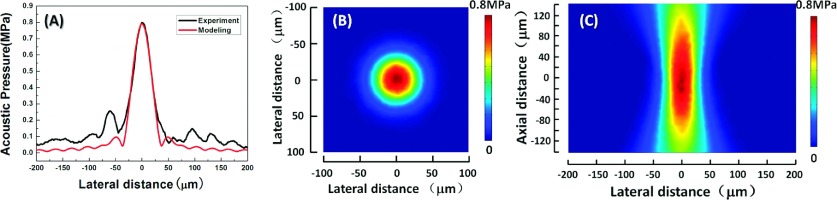
(A) amplitude of acoustic pressure at the focal plane (the black and red solid lines represent the experimental and simulation results, respectively); (B) pressure field distribution (x–y plane) at a frequency of 50 MHz; (C) pressure field distribution (x–z plane) at a frequency of 50 MHz.

**FIG. 4.**
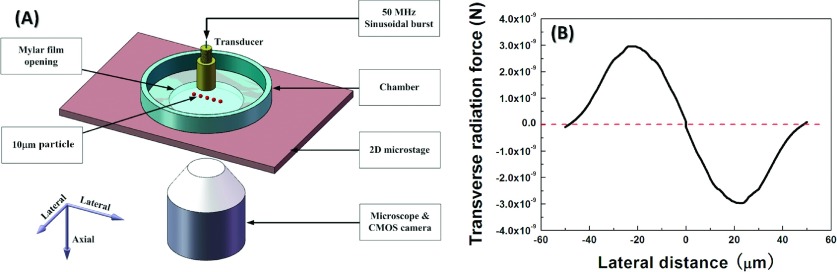
(A) The configuration used in the SBAT trapping experiment; (B) plot of calculated transverse radiation force against the horizontal position of the center within a lateral distance (the red dashed line shows transverse radiation force = 0).

**FIG. 5.**
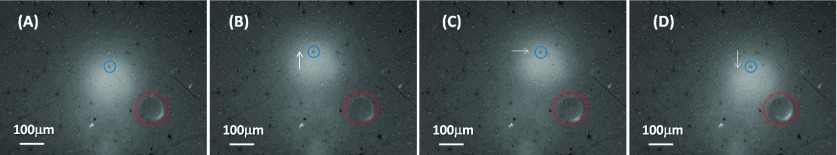
A single 10 μm polystyrene particle trapped and manipulated using an SBAT based on a hydrothermal PZT thick film. The polystyrene particle was trapped (A) and moved in the different directions: (B) Up, (C) Right, (D) Down. (Multimedia view) [URL: http://dx.doi.org/10.1063/1.4943492.1]10.1063/1.4943492.1
